# A qPCR-Based Tool to Diagnose the Presence of Harmful Cyanobacteria and Cyanotoxins in Drinking Water Sources

**DOI:** 10.3390/ijerph14050547

**Published:** 2017-05-20

**Authors:** Yi-Ting Chiu, Yi-Hsuan Chen, Ting-Shaun Wang, Hung-Kai Yen, Tsair-Fuh Lin

**Affiliations:** 1 Department of Environmental Engineering, National Cheng Kung University, Tainan 70101, Taiwan; ostrich_0121@hotmail.com (Y.-T.C.); stevenchen0937@gmail.com (Y.-H.C.); coralmeteor@gmail.com (T.-S.W.); 2 Department of Biological Science and Technology, Meiho University, Pingtung 91202, Taiwan; hkyen@meiho.edu.tw

**Keywords:** cyanobacteria, *Microcystis*, microcystins, *Cylindrospermopsis*, cylindrospermopsin, duplex qPCR

## Abstract

Harmful cyanobacteria have been an important concern for drinking water quality for quite some time, as they may produce cyanotoxins and odorants. *Microcystis* and *Cylindrospermopsis* are two common harmful cyanobacterial genera detected in freshwater lakes and reservoirs, with microcystins (MCs) and cylindrospermopsin (CYN) as their important metabolites, respectively. In this study, two sets of duplex qPCR systems were developed, one for quantifying potentially-toxigenic *Microcystis* and *Microcystis*, and the other one for cylindrospermopsin-producing cyanobacteria and *Cylindrospermopsis*. The duplex qPCR systems were developed and validated in the laboratory by using 338 samples collected from 29 reservoirs in Taiwan and her offshore islands. Results show that cell numbers of *Microcystis* and *Cylindorspermopsis* enumerated with microscopy, and MCs and CYN concentrations measured with the enzyme-linked immuno-sorbent assay method, correlated well with their corresponding gene copies determined with the qPCR systems (range of coefficients of determination R^2^ = 0.392−0.740). The developed qPCR approach may serve as a useful tool for the water industry to diagnose the presence of harmful cyanobacteria and the potential presence of cyanotoxins in source waters.

## 1. Introduction

Lakes and reservoirs are important drinking water sources in Taiwan and many other countries. Due to improper management of nutrients in their watersheds, many reservoirs are facing the risk of eutrophication, increasing the possibility of cyanobacterial blooms in the source waters [1]. Cyanobacteria have been reported to produce a wide variety of chemically unique secondary metabolites, such as hormones, antibiotics, allelochemicals, toxins, and taste and odor (T&O) compounds [2]. Among these metabolites, cyanotoxins may cause illness or death of domestic animals and humans when they are exposed to water contaminated with high cyanobacterial concentrations [3]. Microcystins (MCs) and cylindrospermopsin (CYN) are two well-known cyanotoxin groups found in the reservoirs of Taiwan [4,5,6] and the world [7,8,9,10,11]. MCs have been linked to the increased incidence of primary liver cancer [3,12,13] and acute cases of poisoning in humans and other animals [14]. CYN has been reported to be not only a general cytotoxin but also hepatotoxic and neurotoxic [15,16,17], and has also been linked to the death of domestic animals [18]. Therefore, detection of these two cyanotoxins is an important issue to safeguard drinking water quality.

MCs have been reported to be produced by several planktonic cyanobacterial genera, including *Anabaena* [19], *Anabaenopsis* [20], *Aphanocapsa* [21], *Microcystis* [22], *Nostoc* [23], *Oscillatoria* [24], and *Planktothrix* [25]. However, *Microcystis* spp. are the most commonly detected toxigenic cyanobacteria in the reservoirs in Taiwan [6]. CYN has been reported to be produced by several species, including *Anabaena bergii* [26], *Anabaena lapponica* [27], *Aphanizomenon gracile* [28], *Aphanizomenon flos-aquae* [29], *Aphanizomenon ovalisporum* [30,31,32], *Cylindrospermopsis raciborskii* [8,26,33,34], *Raphidiopsis curvata* [35], and *Umezakia natans* [36]. However, *C. raciborskii* has been considered to be a major problem for water management on a global scale [37].

When high concentrations of cyanobacteria are present or expected to be present in source water, such as cyanobacterial blooms, an estimation of the risks associated with cyanotoxins is always very important for water utilities. Conventionally, the procedures to identify such risks includes a collection of samples, delivery of these samples to a central laboratory, and enumeration of cyanobacterial cells that are potentially toxigenic [38]. Normally, more than 24 h is required to complete the process and obtain the analytical results. During this time, the contaminated water may have already entered the tapwater distribution systems for consumers. Furthermore, field studies show that the reliability of risk estimation based simply on microscopic enumeration is often lower because toxic and non-toxic strains of the same cyanobacterial species often coexist in the environment [39,40,41,42,43]. In addition, taxonomical identification of cyanobacteria using microscopy is a time-consuming process and requires well-trained professionals to perform the analysis [43,44,45]. Therefore, a rapid method to quickly provide information about the potential risks of cyanotoxins and toxigenic cells would be very useful for water utilities to trigger their response actions for episodes of cyanobacteria outbreaks in source waters.

In the last two decades, quantitative real-time polymerase chain reaction (qPCR)-based molecular techniques [4,9,45,46,47] and the enzyme-linked immuno-sorbent assay (ELISA) method [48,49] have been successfully developed and applied to detect toxigenic genes and cyanotoxins, respectively. Both of these techniques have quick turn-around times and are capable of multiple sample analyses in a single run. To date, qPCR systems developed to monitor cyanotoxins are mostly uniplex, with limitation to a single genetic target [9,47]. However, toxic cyanobacterial blooms are usually complex, often involving more than one cyanotoxin [11,50,51], substantiating the importance of detecting multiple producers at the same time. Therefore, multiplex-qPCR, capable of amplifying several different targets in a single reaction well, may provide a simple approach to simultaneously detect multiple target genes. Although these methods have been well documented, most studies are limited to the monitoring of one reservoir/lake and with limited sample size [5,9,10,11,45,46,47,50,52,53,54,55,56,57]. In applying multiplex-qPCR systems, inter-influence of gene abundance on the detection has been reported by only four studies [51,58,59,60], in which less abundant genes were not detected due to the ample presence of other targeted genes. However, only limited information is available for the gene concentration ranges that may cause the inhibition.

The four aims of this study are to develop a qPCR system to detect the cyanobacteria that produce two common cyanotoxins, microcystins and cylindrospermopsin, to test method reliability, to apply the method in field monitoring, and to compare the data with conventional methods for cell numbers and cyanotoxin concentrations. Two duplex qPCR systems were first developed, one for quantifying *Microcystis* and potentially-toxigenic *Microcystis*, and the other for *Cylindrospermopsis* and cylindrospermopsin-producing cyanobacteria. The developed methods were then tested for the influence of DNA concentration on detection, and then applied in the monitoring of the samples collected from 29 drinking water reservoirs throughout Taiwan. Finally, the data measured by qPCR were then compared with cell data and cyanotoxin data, which were determined by microscope method and by ELISA, respectively.

## 2. Materials and Methods

### 2.1. Cyanobacterial Strains and Culturing

Two cyanobacterial strains, *Microcystis aeruginosa* and *Cylindrospermopsis raciborskii*, are used in this study. *Microcystis aeruginosa* PCC7820 strain, a producer of microcystins [61], was purchased from the Pasteur Culture Collection Center (Paris, France), and was used in the preparation of qPCR standard curves for both total *Microcystis* cells and potentially-toxigenic *Microcystis* cells. *Cylindrospermopsis raciborskii* CYP026J strain, a producer of cylindrospermopsin isolated by the Australian Water Quality Centre culture collection (AWQC, Australia), was used for the qPCR standard curves for both total *Cylindrospermopsis* cells and cylindrospermopsin-producing cyanobacteria. Both strains were grown in ASM medium [62] at 25 °C and under a 12 h/12 h light/dark cycle with 19.8 μmol m^−2^ s^−1^ of light intensity.

### 2.2. Study Sites

Water samples were collected from 29 different drinking water reservoirs (DWRs) located throughout the main island of Taiwan and its two off-shore islands, Kinmen Island and Matsu Island. The sampling locations for all the studied reservoirs were all near the water intakes of their associated water treatment plants. The locations of the studied DWRs are shown in Supplementary Materials Figure S1.

### 2.3. Cell Enumeration

Cyanobacteria cells were enumerated following the procedures prescribed in the Standard Method 10200F [63]. In brief, the water samples were fixed with Lugol’s solution (1% by volume) immediately after sampling and stored in the dark until enumeration. For cell counting, water samples were placed into a 1 mL Sedgwick-Rafter chamber (Pyser-SGI Ltd., Kent, UK) and allowed to settle for 30 min. The spherical *Microcystis* cells were then counted under a microscope (BX51, Olympus, Tokyo, Japan) at ×400 magnification. For the filamentous *Cylindrospermopsis*, the cross walls between cells were often indistinct, making this measurement imprecise [43]. Thus, the cells were enumerated by their average cell number per unit length of trichome following the protocol reported in Marbun et al. [5].

### 2.4. Extraction of DNA from Cyanobacterial Cells

To extract DNA from cyanobacterial cells, cyanobacteria-laden water samples (10 mL) were first concentrated using a centrifuge (Hettich Mikro 20 Microfuge, Biotech Equipment, San Francisco, CA, USA) rotating at 16,060 rcf for 5 min. After centrifugation, the supernatant was removed and the cyanobacterial pellets remaining were re-suspended with 5 mg/mL of Lysozyme (Invitrogen™, Life Technologies, Carlsbad, CA, USA), and followed by a freezing-thawing process that was performed three times using liquid nitrogen and a 37 °C water bath for the lysis of cyanobacterial cells [64]. Then, DNA was extracted from the lysed cyanobacterial samples using Illustra™ triplePrep kit (GE Healthcare, Hatfield, UK), following the protocol provided by the manufacturer.

### 2.5. Duplex qPCR System

Each of the developed duplex qPCR systems was performed individually and separately with a qPCR device (Smart Cycler^®^II; Cepheid, Sunnyvale, CA, USA). The first system helps differentiate potentially-toxigenic *Microcystis* cells from that of total *Microcystis* cells, while another system helps distinguish cylindrospermopsin-producing cyanobacteria from that of total *C. raciborskii* cells.

For the *Microcystis* system, primer and probe set Micr184F/Micr431R/Micr228 was used to target the 16S rRNA region unique to *Microcystis* cells [65], while *mcy*B#04F/*mcy*B#04R/*mcy*B#04 were used to specifically target the microcystin synthetase (*mcy*B) region unique to potentially-toxigenic *Microcystis* cells [4]. Detailed information of the primer and probe sets were summarized in Table S1. During analysis, 2 µL of extracted DNA was mixed with 12.5 µL of 2× Rotor-Gene Multiplex PCR Master Mix kit (Qiagen, Hilden, Germany) and 0.1 μM of each primer and probe, with the volume being made up to 25 µL using sterile deionized water. The qPCR protocol includes pre-incubation at 95 °C (300 s), and a 40-cycle of 2-step amplification of denaturation (95 °C, 15 s) and annealing/extension (60 °C, 30 s). At the end of the extension, the fluorescence intensity was recorded at 519 nm for FAM and at 561 nm for Cy3. The threshold cycle (Ct) values were determined using Smart Cycler^®^ software version 2.0 (Cepheid, Sunnyvale, CA, USA).

For identification of *Cylindrospermopsis*, primer and probe set m4/k18/*pks* is efficient for targeting the polyketide synthase (*pks*) region of cylindrospermopsin-producing cyanobacteria [26,45,66], while cyl2/cyl4/*rpo*C1 helps to specifically target the *rpo*C1 region unique to *C. raciborskii* [45,67] (Table S1 (SM)). Also, 0.1 μM of each primer and probe was used during analysis. The qPCR protocol includes a pre-incubation at 95 °C (300 s), and a 40-cycle of 2-step amplification of denaturation (95 °C, 15 s) and annealing/extension (60 °C, 45 s). At the end of the extension, the fluorescence intensity was recorded at 665 nm for Cy5 and at 615 nm for Texas Red. The Ct values were calculated by Smart Cycler^®^ software.

The final reaction mixtures of both uniplex and duplex qPCR systems were optimized by testing the primer and probe concentrations. The primer and probe concentrations corresponding to the lowest Ct and highest fluorescent intensity, respectively, were chosen. Similar Ct’s (difference < 1) were obtained for all the four targeted genes in both uniplex and duplex qPCR systems in this study.

### 2.6. PCR Amplification, Cloning, and Standard Curves

The PCR product of the 16S rRNA region unique to *Microcystis* cells and the *mcy*B region were amplified from the extracted DNA of *M. aeruginosa* PCC7820 strain using the primer sets of Micr184F/Micr431R and *mcy*B#04F/*mcy*B#04R, respectively. In a similar fashion, the PCR product for the *rpo*C1 region and the *pks* region was amplified from the extracted DNA of *C. raciborskii* CYP026J strain using the primer sets of cyl2/cyl4 and m4/k18, respectively.

Sterile deionized water was then added to a PCR mixture, which consisted of 5 µL of the extracted DNA solution, 2 µL of 10× Ex Taq™ buffer, 200 µM of dNTP mix, 0.2 µM of the forward and reverse primers, and 0.1 µL of TaKaRa Ex Taq™ DNA polymerase (Code No. RR01A, Takara Biotechnology, Kusatsu, Japan), such that the volume totalling 50 µL was then placed in a 96-well thermal cycler (C1000™, Bio-Rad Corp., Hercules, CA, USA). The PCR experiment was executed with a pre-incubation at 95 °C (5 min), and a 30-cycle of 3-step amplification of denaturation (98 °C, 10 s), annealing (55 °C, 30 s), and extension (72 °C, 1 min), then a final extension at 72 °C for 10 min.

Then, four PCR products were cloned by using a commercial kit (TOPO^®^ TA Cloning^®^, Invitrogen™, Life Technologies, Carlsbad, CA, USA) following the suggested protocol. The plasmid DNAs from cloning were used to develop the standard curve between the Ct values and the copy numbers. Longer sequences of the target region from plasmid DNAs were amplified by using the universal M13 primers to avoid the possibility of mispaired nucleotides at the ends of the target genes. The M13 PCR product was quantified by measuring the fluorescence intensity with PicoGreen^®^ dsDNA Reagent and Kits (Quant-iT™, Invitrogen™, Life Technologies, Carlsbad, CA, USA). The DNA copy number (copies/μL) was then calculated by dividing the DNA concentration (g/μL) by the molecular weight (g/mole) of the targeted gene and then multiplied by 6.02 × 10^23^ copies/mole [9].

Series of 10-fold dilutions of M13 PCR product of the target genes were prepared (10^2^ to 10^8^ copies/reaction), and the Ct values were plotted against the logarithm of the copy number of the target genes with an amplification efficiency (E) of 100% ± 10%, where E = 10^−1/S^ − 1 (S is the slope of the logarithm regression).

### 2.7. Test of Inhibition on Gene Detection in Duplex Systems

To determine the concentration ratios causing the inhibition, a set of experiments were conducted with different targeted gene concentration ratios for the duplex systems. In these experiments, standard genes of the four studied targets, 16S rRNA, *mcy*B, *pks*, and *rpo*C1, were prepared with varying amounts between 10^3^ and 10^7^ copies/reaction. The prepared standard genes were then measured with the two duplex qPCR systems for *Microcystis* and *Cylindrospermospis*.

### 2.8. Detection of Cyanotoxins

Cyanotoxins in the samples were detected using two commercial ELISA Kits, PN 520012 and PN 522011, both from Abraxis, US for MCs and CYN, respectively [49]. Samples were collected from DWRs then prepared to detect both total cyanotoxins (cell bound + dissolved phase) and dissolved cyanotoxins. For the unfiltered samples, the cyanobacterial cells were ruptured using liquid nitrogen treatments, similar to DNA extraction, to release cell-bound cyanotoxins and then filtered with the 0.22 μm nylon syringe filter to remove cell debris. Dissolved cyanotoxins were separated from cell-bound cyanotoxins using a 0.22 μm nylon syringe filter (Advangene, Lake Bluff, IL, USA). The unfiltered and filtered samples were analyzed for total MCs/CYN and dissolved MCs/CYN, respectively. Following the protocol provided by the manufacturer, 50 μL of the pre-treated MCs/CYN-laden water sample were analyzed. An ELISA reader (Multiskan FC, Thermo Scientific, Vantaa, Finland) was employed to measure the absorbance. The calibration curves were constructed using standard MCs/CYN provided with the ELISA kit.

### 2.9. Statistical Analysis

The field data obtained in this study were analyzed for the correlation between cell enumeration, toxin concentrations, gene copies and cell equivalents of the samples. Although the gene copies in *Microcystis* and *Cylindrospermopsis* may vary with growth status, species, and environmental conditions [68,69], in this study the cell equivalents for the two cyanobacteria were obtained by linking the gene copies of the two standard strains with their cell numbers under the logarithmic growth phase. The calculation of 95% prediction intervals for the linear regressions followed that of Chiu et al. [64]. The regression analysis was conducted with SPSS Statistics 17.0 software (IBM, New York, NY, USA). A statistical software from Minitab (Minitab^®^, State College, PA, USA), was further used to compare the slope coefficients of different regression lines. The slopes are considered to be statistically significantly different when a P value is less than or equal to 0.025 with two-tailed 95% confidence intervals. Another statistical method, the Chow test [70], was also used to test the similarity of the regression coefficients obtained for different data.

## 3. Results and Discussion

### 3.1. Method Development for Quantification of Duplex qPCR Systems

#### 3.1.1. Standard Curves

**Figure 1:**
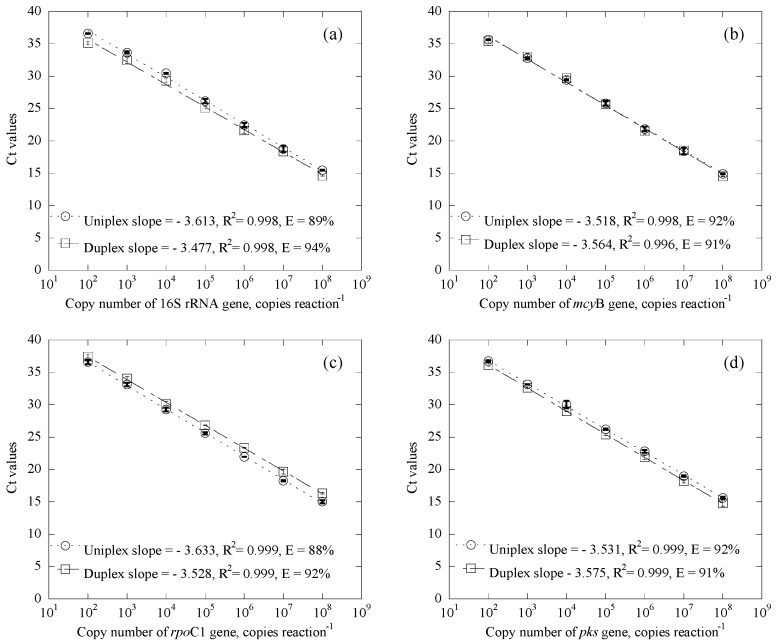
Duplex and uniplex qPCR standard curves for Microcystis and Cylindrospermopsis systems, where (**a**) and (**b**) are for the duplex system of the 16S rRNA gene and mcyB gene, (**c**) and (**d**) are for the duplex system of the rpoC1 gene and pks gene, ◯ represents the results from the uniplex system, ☐ represents the results from the duplex system, and Error bars represent the standard deviation of 2 replicates.

In a comparison of qPCR standard curves for *Microcystis* and *Cylindrospermopsis* systems, results clearly demonstrate that the calibration curves obtained from the duplex quantification system are almost identical to those for the uniplex system, suggesting the equal performance of both systems (Figure 1). The calibration curves of duplex systems for the 16S rRNA gene and *mcy*B gene, as well as the *rpo*C1 gene and *pks* gene, were linear from 10^2^ to 10^8^ copies/reaction with high correlation coefficients, with R^2^ values at 0.998, 0.996, 0.999, and 0.999, respectively. In addition to this, amplification efficiencies of the 16S rRNA gene, *mcy*B gene, *rpo*C1 gene and *pks* gene were 94%, 91%, 92% and 91%, respectively. Such relatively high correlation coefficients and amplification efficiencies suggest that the analytical protocol is reasonable.

#### 3.1.2. Influence of Gene Abundance in the Duplex Systems

To test the applicability of the duplex qPCR systems for field monitoring, the systems were tested in Tai-Hu Reservoir (THR), Kinmen, Taiwan. In the meantime, the samples were also analyzed with the uniplex qPCR systems. The duplex system results were compared with their respective uniplex system for the detection of the targeted cyanobacteria. The data, which are shown in Tables S2 and S3 for *Microcystis* and *Cylindrospermosis*, respectively, were collected and analyzed from February 2013 to August 2016. Among the 13 samples analyzed for both *Microcystis* and *Cylindrospermopsis*, most results from the duplex systems closely matched with those for a uniplex system. However, a discrepancy was found for three samples collected in February 2013, November 2013 and March 2014, which tested specifically for *Cylindrospermopsis* (Table S3 and Table 1). Although high concentrations of CYN were measured, only the uniplex system was able to capture both *rpo*C1 and *pks* genes, while the duplex system failed to detect the *pks* gene (Table 1). The failure of the duplex system may be caused by differences in primer concentration, probe concentration, polymerase/dNTP/buffer concentration, annealing/extension time and amount of template DNA [71,72,73,74]. Similarly, previous studies have also shown that the amount of template DNA affects its detection in multiplexed systems [51,58,59,60], where the detection of low concentration genes was inhibited by the high concentration ones. In this study, the Ct values for the *rpo*C1 gene in these three uniplex-based samples were 9–10 cycles smaller than those for the *pks* gene, suggesting that abundance of the *pks* gene in the sample was much lower (Table 1). This is in accordance with those reported in Wang and Mustapha [58], Hyeon et al. [59], Dai et al. [60] and Te and Gin [51] that detection of a lower gene concentration was inhibited in multiplexed systems.

To determine the concentration ratios causing the inhibition, the results clearly show that, for the duplex *Microcystis* system when *mcy*B gene copies were 10^2^ times higher than that of 16S rRNA gene, the system fails to detect the less abundant gene (Table 2). In contrast, detection of the *mcyB* gene was not affected even when 16S rRNA gene abundance was 10^4^ times higher. Fortunately, since the abundance of the *mcy*B gene, which is a gene that identifies a subset of *Microcystis* cells that are potentially toxigenic [4], should always be less than or equal to the total number of *Microcystis* cells as identified via the 16S rRNA gene [65], the issue of false negative results for 16S rRNA would not be the problem in the duplex *Microcystis* system (Table S2).

For the duplex *Cylindrospermopsis* system, when *pks* gene copies were 10^3^ times higher than that of *rpo*C1 gene, no *rpo*C1 gene will be detected. On the other hand, *pks* gene was not detected if the abundance of *rpo*C1 gene copies were 10^2^ times higher. Since the *pks* gene primer was designed for cylindrospermopsin-producing cyanobacteria [26,45,66], and the *rpo*C1 gene primer was targeted specifically on *C. raciborskii* [45,67], those detected by the *pks* gene primer are not necessary all detected by the *rpo*C1 gene primer, as some cyanobacteria such as *Anabaena bergii* [26], *Anabaena lapponica* [27], *Aphanizomenon gracile* [28], *Aphanizomenon flos-aquae* [29], *Aphanizomenon ovalisporum* [30,31,32], *Raphidiopsis curvata* [35], and *Umezakia natans* [36] may also produce cylindrospermopsin. Therefore, in applying this duplex *Cylindrospermopsis* system, if either gene is detected at >10^5^ copy/reaction and the other one is not detected, the uniplex system should be used to confirm the presence of the second gene.

Attempts have been made to improve the detection of the lower abundant targeted genes, including primer concentration, probe concentration, polymerase/dNTP/buffer concentration and annealing/extension time (Figure S2 and Table S4). However, none of them can reduce the influence on the inhibition of the detection. Further studies are needed to improve the inhibition of detection for the lower abundant targeted genes in multiplexed systems.

### 3.2. Applications of the Duplex Systems in Reservoir Water Samples

**Figure 2:**
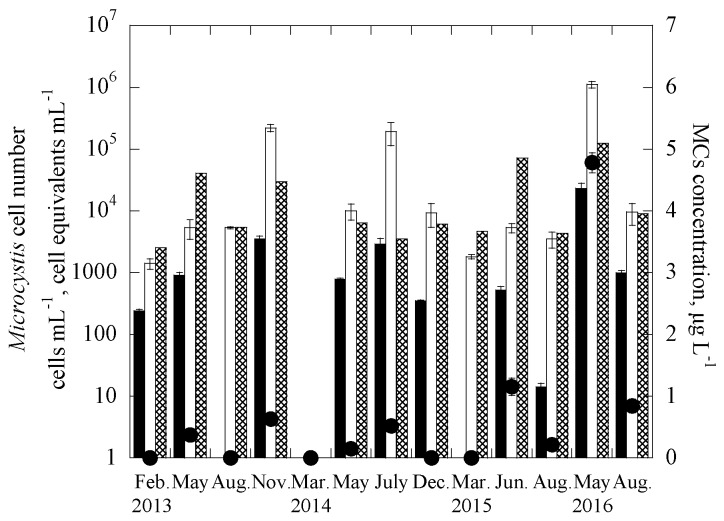
Time course of 16S rRNA gene, *mcy*B gene and microcystins (MCs) concentrations for Tai-Hu Reservoir (THR) in the duplex Microcystis system, where ■ is the cell equivalent of the *mcy*B gene, ☐ is the cell equivalent of the 16S rRNA gene, ⊠ is the cell abundance using microscopy, and ● is the MCs concentration. Error bars represent the standard deviation of 2 replicates.

**Figure 3:**
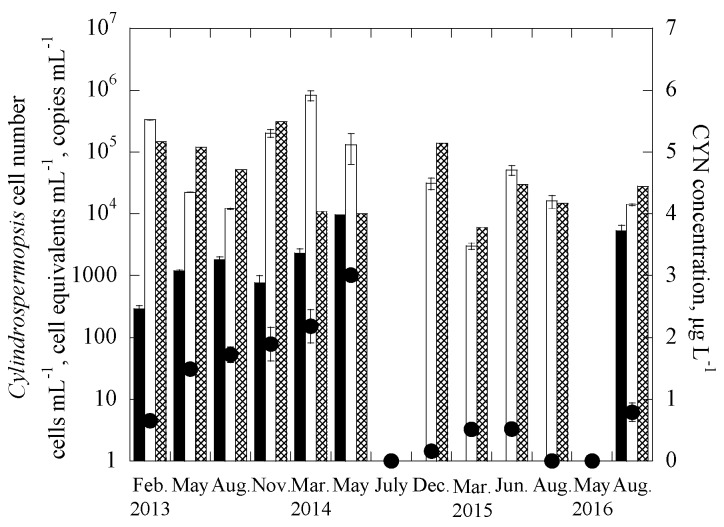
Time course of *rpo*C1 gene, *pks* gene and cylindrospermopsin (CYN) concentrations for THR in the duplex *Cylindropsermopsis* system, where ■ are the *pks* gene copies, ☐ is the cell equivalent of the *rpo*C1 gene, ⊠ is the cell abundance using microscopy, and ● is the CYN concentration. Error bars represent standard deviation of 2 replicates.

The two duplex systems were further applied to quantify the targeted genes in 338 samples collected between February 2013 and August 2016 from 29 reservoirs throughout Taiwan, consisting of 10 drinking water reservoirs (DWRs) on the main island of Taiwan, 10 DWRs in the Kinmen islands, and 9 DWRs in the Matsu islands (Figure S1). In addition, the samples were also analyzed for the two cyanotoxins, MCs and CYN, and enumerated for cell numbers of the two targeted cyanobacteria. Figure 2 and Figure 3 show the monitoring results for one of the studied reservoirs, THR in Kinmen islands, for *Microcystis* and MCs and *Cylindrospermopsis* and CYN, respectively. It is noted that for the reservoir samples, the gene abundance (copies-mL^−1^) was transferred to the cell equivalents concentration (cells-mL^−1^) through the calibration curves constructed for the laboratory cultures [65], as shown in Figure S3. Based on this, the total number of *Microcystis* cell equivalents, potentially-toxigenic *Microcystis* cell equivalents, and the total number of *Cylindrospermopsis* cell equivalents in a sample can be estimated, from the abundance of the 16S rRNA gene, *mcy*B gene, and *rpo*C1 gene, respectively. It is noted that the primer and probe set for the *pks* gene is efficient for targeting the polyketide synthase (*pks*) region of cylindrospermopsin-producing cyanobacteria, including *Cylindrospermopsis raciborskii*, *Aphanizomenon ovalisporum*, *Raphidiopsis curvata*, *Anabaena bergii* and *Oscillatoria sp.* [26,45,66]. As the genome copies of cyanobacteria may vary in different genera [68,69], the gene copies were not transferred into cell equivalent of cylindrospermopsin-producing cyanobacteria in the samples.

Results showed that the cell-equivalent concentrations of field samples determined with duplex qPCR methods, 16S rRNA gene for *Microcystis* and *rpo*C1 gene for *Cylindrospermopsis*, were mostly similar to cell concentrations enumerated with microscopy, with only 5 of the 13 Kinmen samples having a difference of more than one order of magnitude (Figure 2 and Figure 3). In natural water, *Microcystis* cells often grow in colonies [75,76], while *Cylindrospermopsis* trichomes are 50–250 μm long, and slightly constricted or not constricted at the cross walls between cells [43]. Colonies and trichomes may prohibit the uniform distribution of *Microcystis* and *Cylindrospermopsis* cells in the water samples, making the cell enumeration and gene detection difficult. The discrepancy observed for the two analytical methods, the qPCR and microscope methods may be caused by the non-uniform distribution of the cells in natural water samples.

The increase of the cell-equivalent concentration of toxigenic *microcystis* (based on the *mcy*b gene) and copy number of cylindrospermopsin producers (based on the *pks* gene) follow an increase in the concentration of MCs and CYN, respectively. Note that for each particular toxin, the data obtained for two of the sampling dates did not follow the trends. Figure 2 showed that, for the duplex *microcystis* system, only *mcy*b gene and no MCs were detected in the samples collected in February 2013 and May 2014; while in Figure 3, for the duplex *cylindrospermopsis* system, only CYN and no *pks* genes were detected in the 2 samples collected in December 2014 and August 2015. The discrepancy between the two methods, gene detection, and ELISA, may be attributed to a few reasons. First, detection of *mcy*b and *pks* genes represents the presence of potential producers, but not necessarily the presence of products (toxins) [77,78,79,80]. It is possible that some of the producers were not producing the cyanotoxins during the sampling. Although naked DNA from lysed cells might be present in the samples, in the current experimental protocol, the naked DNA was expected not to be extracted using centrifugation [64]. Thus, it is possible that in the *Cylindrospermopsis* system, the lysed cells or extremely low cell concentrations may lead to no detection of the *pks* gene in the samples. However, the released CYN was still measurable.

In Figure 2, the results also demonstrate that abundance of *Microcystis* (detected with 16S rRNA gene) was always higher than that of potentially toxigenic species (*mcy*B gene). Previous research demonstrated that toxic and non-toxic strains of the same cyanobacterial species often coexist in the environment [39,40,41,42]. The results of this study show that 1.53–17.14% of *Microcystis* cells detected were capable of producing MCs, confirming the observations of previous field studies that not all strains of *Microcystis* found in natural samples are capable of producing toxins [65,78].

### 3.3. Correlations among Cell Numbers, Toxin Concentrations, Gene Copies and Cell Equivalents of the Samples

**Figure 4:**
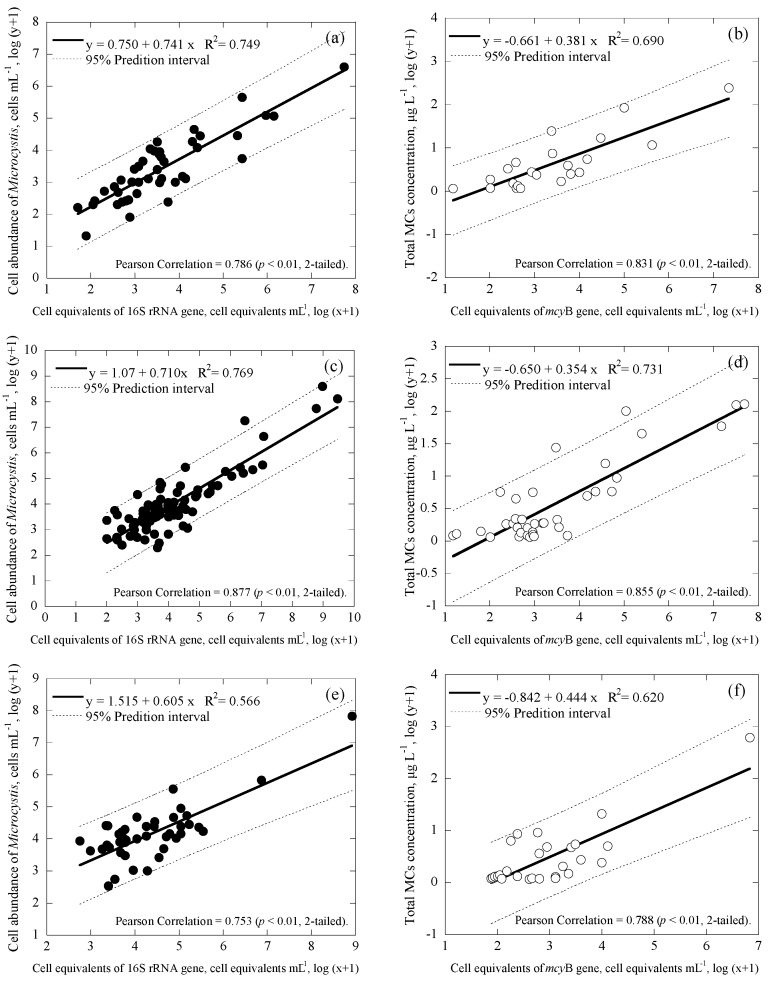
Correlation between cell abundance, toxin concentrations and cell equivalents in the duplex *Microcystis* system with 95% prediction interval (broken line), where ● represents cell equivalents of 16S rRNA gene, ◯ represents cell equivalents of *mcy*B gene, (**a**) and (**b**) are for all the data from Taiwan main island (number of samples, *n* = 44 and 22), (**c**) and (**d**) are for Kinmen islands (*n* = 86 and 38), and (**e**) and (**f**) are for Matsu islands (*n* = 43 and 27).

**Figure 5:**
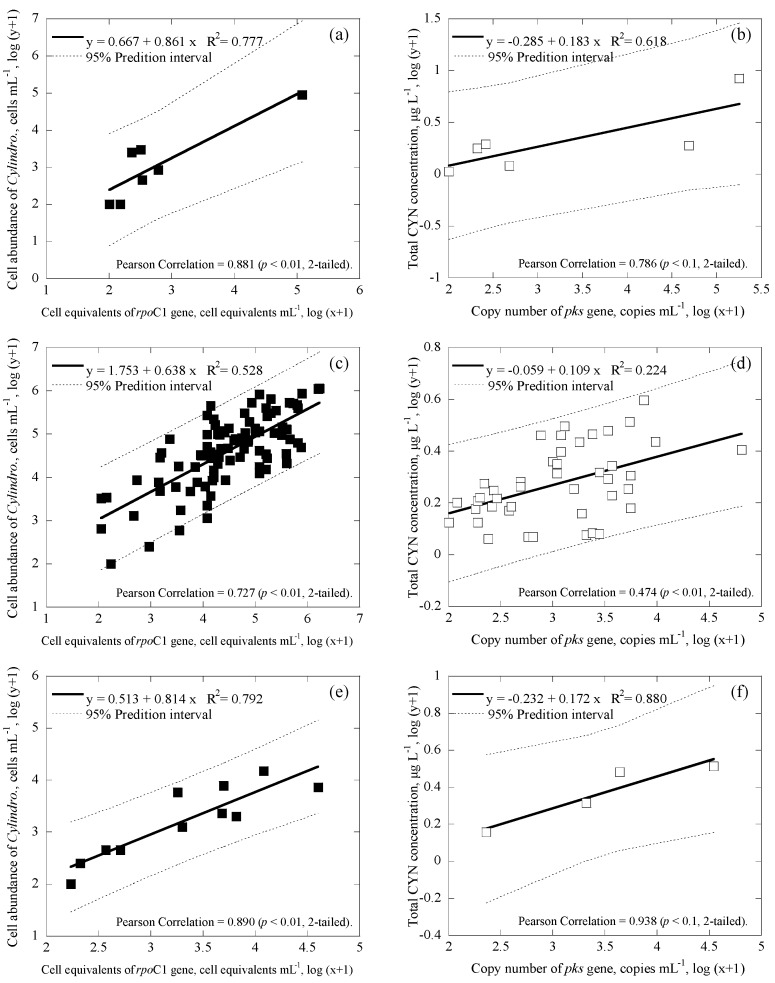
Correlation between cell abundance, toxin concentrations and cell equivalents in the duplex *Cylindrospermopsis* system with 95% prediction interval (broken line), where ■ represents cell equivalents of *rpo*C1 gene, ☐ represents the copy number of *pks* gene, (**a**) and (**b**) are for all the data from Taiwan main island (number of samples, *n* = 7 and 6), (**c**) and (**d**) are for Kinmen islands (*n* = 91 and 43), and (**e**) and (**f**) are for Matsu islands (*n* = 11 and 4).

*Microcystis* and *Cylindrospermopsis* data collected from all the reservoirs were separated into three main groups based on differences in geographic location: Taiwan main island (Figure 4a,b for *Microcystis*; Figure 5a,b for *Cylindrospermopsis*); Kinmen islands (Figure 4c,d for *Microcystis*; Figure 5c,d for *Cylindrospermopsis*); and Matsu islands (Figure 4e,f for *Microcystis*; Figure 5e,f for *Cylindrospermopsis*). Analyses of both *Microcystis* and *Cylindrospermopsis* were further separated. In order to better understand the effectiveness of this study’s new duplex system, two comparisons were made for *Microcystis* at each geographic location: cell equivalents concentrations (based on the 16S rRNA gene) was compared to the cell abundance (microscopy); potentially toxigenic cell equivalents (*mcy*B gene) was compared to MC concentration (ELISA). The comparisons for *Cylindrospermopsis*: cell equivalents (*rpo*C1 gene) were compared to cell abundance (microscopy); cylindrospermopsin producing the gene copy number (*pks* gene) was compared to CYN concentration (ELISA).

Regardless of geographic location, correlation values between the cell equivalents of total *Microcystis* concentrations obtained via duplex and microscopy were relatively moderate to strong, ranging from R^2^ = 0.566−0.769 (*p* < 0.01; Figure 4a,c,e). Similarly, the correlation value between the cell-equivalent concentration of potentially toxigenic *Microcystis* obtained via duplex and MCs concentration obtained via ELISA was also moderate to strong, ranging from R^2^ = 0.620−0.731 (*p* < 0.01; Figure 4b,d,f). Furthermore, regardless of geographic location, the coefficient of cell abundance compared to conventional methodology remained consistent. Coefficients of cell equivalents of total *Microcystis* concentrations obtained via duplex compared to microscopy ranged from 0.605 to 0.741 (Figure 4a,c,e); while coefficients of cell-equivalent concentrations of potentially toxigenic *Microcystis* obtained via duplex compared to MCs concentrations varied, within an even smaller range of 0.354 to 0.444 (Figure 4b,d,f). This suggests that regardless of differences in environmental factors due to changes in geographic location, slope value remains consistent.

For *Cylindrospermopsis*, the correlation values between the cell equivalents of total *Cylindrospermopsis* concentrations obtained via duplex and microscopy were relatively moderate to strong, ranging from R^2^ = 0.528−0.792 (*p* < 0.01; Figure 5a,c,e). Similarly, the correlation value between the copy number of the cylindrospermopsin producing gene obtained via duplex and the CYN concentration obtained via ELISA was also moderate to strong, ranging from R^2^ = 0.224−0.880 (*p* < 0.01; Figure 5b,d,f). Furthermore, the coefficients of the cell equivalents of total *Cylindrospermopsis* concentrations obtained via duplex compared to microscopy ranged from 0.638 to 0.861 (Figure 4a,c,e); while coefficients of the copy number of cylindrospermopsin producing gene obtained via duplex compared to CYN concentration varied within an even smaller range of 0.109 to 0.183 (Figure 4b,d,f).

During analyses, the methodology developed in this study revealed consistent results, regardless of geographic grouping. In general, results showed that coefficients for linear regression amongst all data were similar, showing that the method in this study is consistent regardless of location. For cell abundance of the total *Microcystis*, comparison of the duplex qPCR method to microscopy (using the Minitab statistical software) showed that, regardless of geographic grouping, there were no significant differences for the three geological locations, with *p* = 0.704, 0.2620, and 0.206 (different if *p* ≤ 0.025 (two-tailed) for 95% confidence interval) for Taiwan main island and Kinmen islands, Taiwan main island and Matsu islands, and Kinmen islands and Matsu islands, respectively. Similar results were also observed for comparison of all other three cell/cyanotoxin to gene correlations for the three geological locations, with *p* ≥ 0.025 for the cases of *mcy*B and MCs concentration, *rpo*C1 and *Cylindrospermopsis* abundance, and pks and CYN concentration.

Several parameters may affect the correlation slope of the target compound to the genes, including toxin production rate and cell quota (toxin concentration per cell equivalents), toxin-producing species, and gene copy of the producing species [64]. Therefore, the correlation equations between cells/cyanotoxins and genes obtained for the three geological locations for both *Microcystis* and *Cylindrospermopsis* systems were further tested for regression stability, using Chow test [68]. The result suggests that the coefficients in all three linear regression models for three geological locations are equal (*p* < 0.01 in Figure 4a,c,e; *p* < 0.05 in Figure 4b,d,f; *p* < 0.001 in Figure 5a,c,e; and *p* < 0.05 in Figure 5b,d,f). Although correlation values and slope values between the duplex qPCR method and conventional methods were moderate to strong, statistical analyses reveal that geological location does not necessarily determine the correlation values and slope values in this study. According to this result, the methodology developed in this study should not be limited by location, but rather applicable to other study areas.

**Figure 6:**
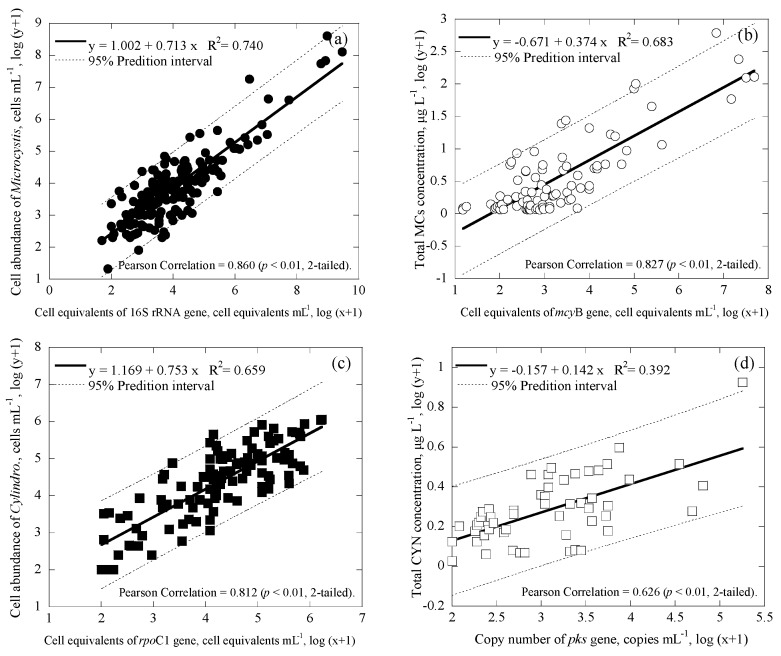
Correlation between cell enumeration, toxin concentrations, gene copies and cell equivalents for all the data with a 95% prediction interval (broken line), where (**a**) is for the cell enumeration of *Microcystis* using microscopy and cell equivalents of the 16S rRNA gene (*n* = 173), (**b**) is for the total MCs concentration and cell equivalents of the *mcy*B gene (*n* = 87), (**c**) is for the cell enumeration of *Cylindropsermopsis* using microscopy and the cell equivalents of *rpo*C1 gene (*n* = 109), and (**d**) is for the total CYN concentration and copy number of *pks* gene (*n* = 53).

In this study, the slopes of correlations obtained for Taiwan main island, Kinmen islands and Matsu islands fall in a narrow range. Therefore, all the data for the three geographical locations were further correlated. Figure 6 demonstrates the good correlations for the data collected from all geographic locations and may provide a simple way to link the data between cell numbers and gene copies, and toxin concentrations and gene copies or cell equivalents, for the two studied cyanobacteria in reservoirs.

In Figure 4, Figure 5 and Figure 6, 95% prediction intervals were also calculated and displayed. As seen, the majority of data (94.7–100% in Figure 4, 95.6–100% in Figure 5, and 93.1–98.1% in Figure 6) fall within the 95% prediction intervals. This may suggest that the correlations developed are able to capture the variations of the tested cyanobacteria and metabolites in the reservoirs studied. As gene results for *Microcystis* and *Cylindrospermopsis* obtained from this study showed somewhat strong correlation values with the cell number information obtained from microscopy and the cyanotoxin concentration from ELISA, respectively, the qPCR-based method may be a better alternative for the monitoring of cyanobacteria and metabolites in natural water. In addition, since the qPCR system is able to distinguish toxic and nontoxic species, the approach has the potential to quickly provide useful information for water utilities to estimate the potential risks of toxins and toxigenic cells in the source waters.

## 4. Conclusions

Two sets of duplex qPCR systems were successfully developed for quantifying two commonly observed harmful cyanobacteria in this study; the first consisted of *Microcystis* and microcystin-producing *Microcystis* using 16S rRNA and *mcy*B, respectively; the second set consisted of *Cylindrospermopsis* and cylindrospermopsin-producing cyanobacteria using *rpo*C1 and *pks* gene, respectively. Field and laboratory tests showed that, in the duplex qPCR systems, presence of a highly abundant targeted gene may inhibit the detection of the other less abundant one. Although *mcyB* gene was detectable even when the concentration of 16S rRNA gene was four orders of magnitude higher, however, for the *Cylindrospermopsis* system, when either *pks* or *rpo*C1 gene copies were 10^2^–10^3^ times higher than the other one, the system would fail to detect the lower abundant gene. Analysis of the samples collected from 29 reservoirs in Taiwan and her offshore islands show good correlation values between cell (equivalent) numbers and gene copies, and between toxin concentrations and gene copies for both *Microcystis* and *Cylindrospermopsis* systems, with R^2^ mostly >0.60 for the reservoirs in three geographical locations. Relatively high coefficients of determination were found for the correlations between *Microcystis* cells and 16S rRNA gene (R^2^ = 0.740), microcystin and *mcy*B (R^2^ = 0.683), *Cylindrospermopsis* cells and *rpo*C1 gene (R^2^ = 0.659), and cylindrospermopsin and *pks* gene (R^2^ = 0.392), for all the samples collected in the reservoirs. The results demonstrate that the developed duplex qPCR systems were able to quantify the targeted cyanobacteria and toxin producing genes. In particular, the obtained gene information correlated well with cell numbers enumerated with microscopy and toxin concentrations measured with ELISA. The qPCR approach may be used as a tool for water utilities to diagnose the potential risk associated with toxigenic cyanobacteria and cyanotoxins in drinking water lakes and reservoirs.

## Figures and Tables

**Table 1:** The interference of gene detection in the duplex *Cylindrospermopsis* system.

Samples	Date		Uniplex (Ct Value)		Duplex (Ct Value)		CYN Concentration (μg/L)
			*pks* Gene	*rpo*C1 Gene	*pks* Gene	*rpo*C1 Gene	
THR	2013	February	38.64 (±0.14) ^1^	27.41 (±0.10)	- ^2^	28.48 (±0.06)	0.65
		November	37.13 (±0.17)	28.27 (±0.17)	-	29.26 (±0.05)	1.89
	2014	March	35.68 (±0.15)	25.91 (±0.05)	-	27.10 (±0.04)	2.18

^1^ Represents standard deviation of 2 replicates; ^2^ - Represents the result < detection limit (Table S1).

**Table 2:** Test of influence on gene detection using different amounts of standard DNA.

Duplex *Microcystis* System				Duplex *Cylindrospermopsis* System			
Copies/Reaction		Ct Value		Copies/Reaction		Ct Value	
*mcy*B	16S rRNA	*mcy*B	16S rRNA	*pks*	*rpo*C1	*pks*	*rpo*C1
10^3^	10^3^	33.52 (±0.42)^1^	32.04 (±0.07)	10^3^	10^3^	32.69 (±0.33)	34.12 (±0.23)
	10^4^	33.41 (±0.57)	30.07 (±0.04)		10^4^	32.70 (±0.28)	30.31 (±0.13)
	10^5^	32.84 (±0.61)	29.95 (±0.37)		10^5^	- ^2^	26.69 (±0.32)
	10^6^	33.40 (±0.08)	21.68 (±0.42)		10^6^	-	23.39 (±0.08)
	10^7^	32.69 (±0.43)	18.29 (±0.26)		10^7^	-	19.99 (±0.04)
10^4^	10^3^	29.91 (±0.01)	32.33 (±0.36)	10^4^	10^3^	29.07 (±0.45)	33.51 (±0.06)
	10^4^	29.75 (±0.41)	29.66 (±0.17)		10^4^	29.05 (±0.16)	30.82 (±0.11)
	10^5^	30.50 (±0.38)	26.03 (±0.06)		10^5^	28.86 (±0.13)	26.62 (±0.62)
	10^6^	30.31 (±0.30)	22.19 (±0.01)		10^6^	-	23.28 (±0.24)
	10^7^	30.01 (±0.29)	18.55 (±0.57)		10^7^	-	19.69 (±0.18)
10^5^	10^3^	26.13 (±0.21)	-	10^5^	10^3^	25.75 (±0.07)	33.29 (±0.15)
	10^4^	26.23 (±0.26)	29.81 (±0.48)		10^4^	25.73 (±0.13)	30.78 (±0.28)
	10^5^	26.12 (±0.40)	25.69 (±0.52)		10^5^	25.43 (±0.08)	27.32 (±0.07)
	10^6^	25.85 (±0.33)	21.92 (±0.10)		10^6^	24.96 (±0.25)	24.16 (±0.13)
	10^7^	25.85 (±0.31)	18.53 (±0.47)		10^7^	-	19.76 (±0.13)
10^6^	10^3^	22.37 (±0.04)	-	10^6^	10^3^	22.05 (±0.28)	-
	10^4^	22.31 (±0.48)	-		10^4^	21.99 (±0.07)	30.59 (±0.20)
	10^5^	22.54 (±0.54)	25.54 (±0.04)		10^5^	22.20 (±0.05)	27.41 (±0.55)
	10^6^	21.88 (±0.62)	21.31 (±0.00)		10^6^	22.39 (±0.13)	23.96 (±0.25)
	10^7^	22.00 (±0.60)	18.18 (±0.73)		10^7^	21.81 (±0.18)	19.87 (±0.13)
10^7^	10^3^	18.88 (±0.13)	-	10^7^	10^3^	18.35 (±0.01)	-
	10^4^	19.03 (±0.06)	-		10^4^	18.44 (±0.11)	-
	10^5^	18.94 (±0.50)	-		10^5^	18.37 (±0.07)	26.91 (±0.31)
	10^6^	18.73 (±0.22)	21.95 (±0.33)		10^6^	18.45 (±0.30)	24.13 (±0.03)
	10^7^	18.78 (±0.20)	18.38 (±0.11)		10^7^	18.24 (±0.13)	19.88 (±0.14)

^1^ Represents standard deviation of 2 replicates; ^2^ - Represents the result < detection limit (Table S1).

## Supplementary Materials

The following are available online at www.mdpi.com/1660-4601/14/5/547/s1, Figure S1: Locations of the studied reservoirs, Figure S2: Tests of inhibition on gene detection caused by different amounts of standard DNA using gel electrophoresis, Figure S3: The relationship between cell enumeration measured with microscopy and gene copy number with qPCR, Table S1: Detailed information of oligonucleotides, Table S2: Monitoring results of *Microcystis* and microcystins for the samples collected from Tai-Hu Reservoir (THR), Table S3: Monitoring results of *Cylindrospermopsis* and cylindrospermopsin for the samples collected from Tai-Hu Reservoir (THR), Table S4: The influence of primer concentration on the inhibition of gene detection, Table S5: Correlation between MCs/CYN concentrations and cell equivalents.
