# Supplementary material for: A qPCR-Based Tool to Diagnose the Presence of Harmful Cyanobacteria and Cyanotoxins in Drinking Water Sources
Source: Int J Environ Res Public Health. 2017 May 20;14(5):547. doi: 10.3390/ijerph14050547 (PMC5451997; doi:10.3390/ijerph14050547)
Supplement: Supplementary file 1 [file ijerph-14-00547-s001.pdf]

Supplementary Materials

A qPCR-based tool to diagnose the presence of harmful cyanobacteria and cyanotoxins in drinking water sources

Yi-Ting Chiu [1], Yi-Hsuan Chen [1], Ting-Shaun Wang [1], Hung-Kai Yen [2] and Tsair-Fuh Lin [1, *]

Captions

Figure S1 – Locations of the studied reservoirs: (a) 10 studied reservoirs in Taiwan, (b) 9 studied reservoirs in Matsu, and (c) 10 studied reservoirs in Kinmen. The Hsin-Shan Reservoir (HSR), Shih-Men Reservoir (SMR), Bao-Shan Reservoir (BSR), Bao-Shan Second Reservoir (BSSR), Liyutan Reservoir (LYTR), Lan-Tan Reservoir (LTR), Nan-Hua Reservoir (NHR), Agongdian Reservoir (AGDR), and Fong-Shan Reservoir (FSR) in Taiwan; the Hou-Wo Reservoir (HWR), Chu-Shui-Wo Lower Dam (CSWLD), Chu-Shui-Wo Upper Dam (CSWUD), Jin-Sha Reservoir (JSR-M), First Jin-Sha Reservoir (FJSR), Sheng-Li Reservoir (SLR), Tsair-Pu-Wo Reservoir (TPWR), Le-Dao-Wo Reservoir (LDWR), and Jhu-Luo Reservoir (JLR) in Matsu; the Rong-Hu Reservoir (RHR), Jin-Sha Reservoir (JSR-K), Tian-Pu Reservoir (TPR), Lan-Hu Reservoir (LHR), Lian-Hu Reservoir (LianHR), Ling-Hu Reservoir (LingHR), Yang-Ming-Hu Reservoir (YMHR), Xi-Hu Reservoir (XHR), Jin-Hu Reservoir (JHR), and Tai-Hu Reservoir (THR) in Kinmen.

Figure S2 – Tests of inhibition on gene detection caused by different amounts of standard DNA using gel electrophoresis, where M represents the DNA marker, N represents the negative control, P5R5, P3R5 and P6R3 represent the concentration of *pks* gene and *rpo*C1 gene with 2 replicates, respectively (P5R5 = 10^5 and 10^5; P3R5 = 10^3 and 10^5; P6R3 = 10^6 and 10^3). (a) is for the duplex qPCR system with primer and probe sets of *pks* gene and *rpo*C1 gene; (b) is for the duplex qPCR system with primer sets of *pks* gene and *rpo*C1 gene (without probes).

Figure S3 – The relationship between cell enumeration measured with microscopy and gene copy number with qPCR, where (a) is for 16S rRNA gene, (b) is for *mcy*B gene, and (c) is for *rpo*C1 gene. Error bars represent standard deviation of 2 replicates.

Table S1 – Detailed information of oligonucleotides.

Table S2 – Monitoring results of *Microcystis* and microcystins for the samples collected from Tai-Hu

Reservoir (THR).

Table S3 – Monitoring results of *Cylindrospermopsis* and cylindrospermopsin for the samples collected from Tai-Hu Reservoir (THR).

Table S4 –The influence of primer concentration on the inhibition of gene detection.

Table S5 – Correlation between MCs/CYN concentrations and cell equivalents.

Table S1 – Detailed information of oligonucleotides

Primer/Probe name	Sequence (5'-3')	product size (bp)	Detection limit (This study)	References
Potentially-toxigenic *Microcystis* cell equivalents (*mcy*B region)				
*mcy*B#04F	TGTGGAGTCTATTTATCCTCTTTCC	95	4.8×10^{1}	Yen *et al.* 2012
*mcy*B#04R	GAGTTTGACTACAATAAATCCCTGAAT		cell equivalents/mL	Yen *et al.* 2012
*mcy*B#04	FAM/CAGGAAGGGATGCTCTTTCA/BHQ_1			Yen *et al.* 2012
Total *Microcyctis* cell equivalents (16S rRNA region)				
Micr184F	GCCGCRAGGTGAAAMCTAA	247	2.6×10^{2}	Rinta-Kanto *et al.* 2005
Micr431R	AATCCAAARACCTTCCTCCC		cell equivalents/mL	Rinta-Kanto *et al.* 2005
Micr228	Cy3/AAGAGCTTGCGTCTGATTAGCTAGT/BHQ_2			Rinta-Kanto *et al.* 2005
Total *Cylindrospermopsis* cell equivalents (*rpo*C1 region)				
cyl2	GGCATTCCTAGTTATATTGCCATACTA	308	1.0×10^{2}	Wilson et al., 2000
cyl4	GCCCGTTTTTGTCCCTTTGCTGC		cell equivalents/mL	Wilson et al., 2000
*rpo*C1	Cy5/TCCTGGTAATGCTGACACACTCG/BHQ_2			Rasmussen et al., 2008
Cylindrospermopsin-producing gene (*pks* region)				
m4	GAAGCTCTGGAATCCGGTAA	422	5.0×10^{2}	Schembri et al., 2001
k18	CCTCGCACATAGCCATTTGC		copies/mL	Fergusson and Saint, 2003
pks	TexasRed/CGGCAGCAACACTCACATCAGT/BHQ_2			Rasmussen et al., 2008

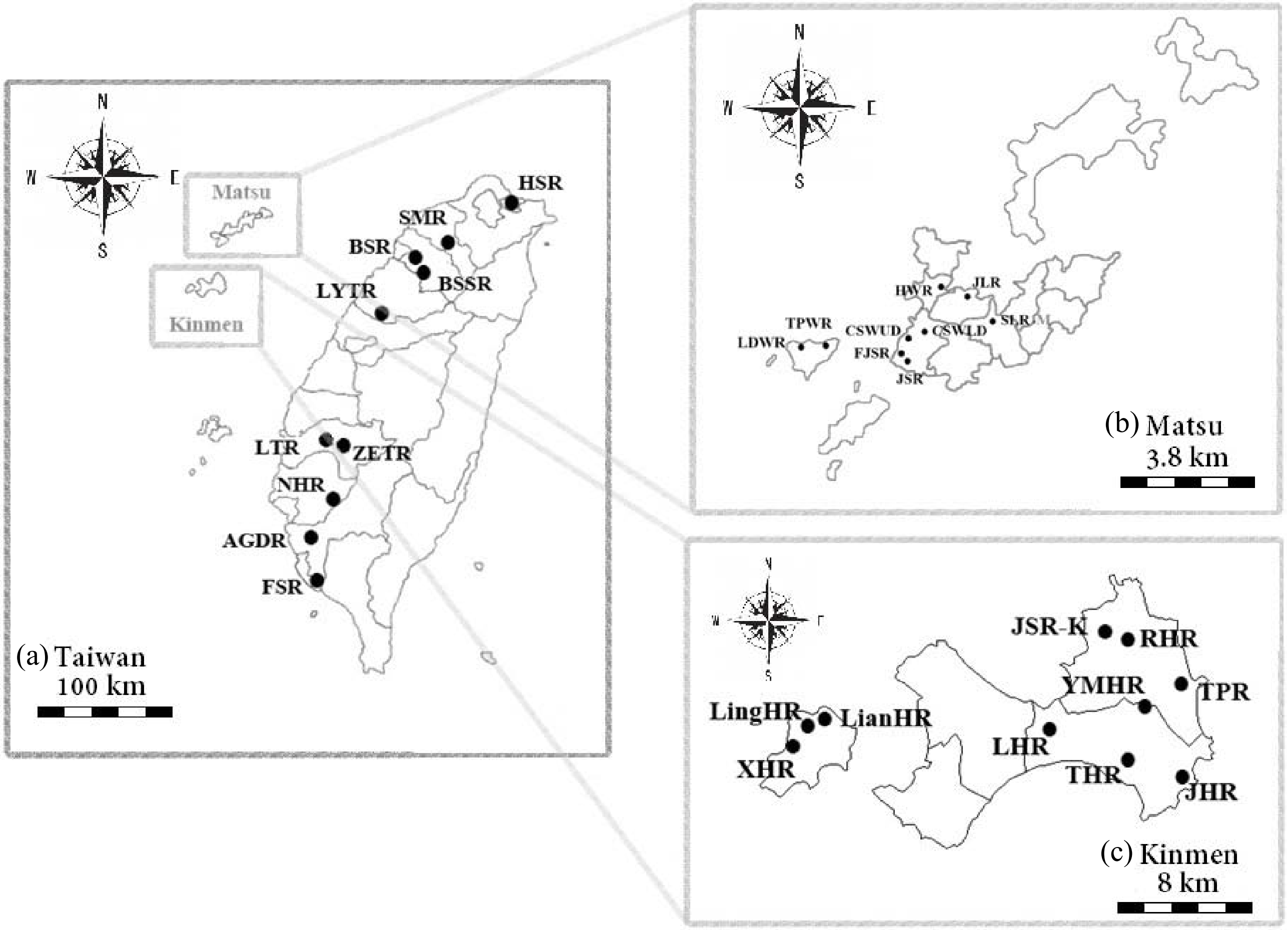

Figure S1 – Locations of the studied reservoirs: (a) 10 studied reservoirs in Taiwan, (b) 9 studied reservoirs in Matsu, and (c) 10 studied reservoirs in Kinmen. The Hsin-Shan Reservoir (HSR), Shih-Men Reservoir (SMR), Bao-Shan Reservoir (BSR), Bao-Shan Second Reservoir (BSSR), Liyutan Reservoir (LYTR), Lan-Tan Reservoir (LTR), Nan-Hua Reservoir (NHR), Agongdian Reservoir (AGDR), and Fong-Shan Reservoir (FSR) in Taiwan; the Hou-Wo Reservoir (HWR), Chu-Shui-Wo Lower Dam (CSWLD), Chu-Shui-Wo Upper Dam (CSWUD), Jin-Sha Reservoir (JSR-M), First Jin-Sha Reservoir (FJSR), Sheng-Li Reservoir (SLR), Tsair-Pu-Wo Reservoir (TPWR), Le-Dao-Wo Reservoir (LDWR), and Jhu-Luo Reservoir (JLR) in Matsu; the Rong-Hu Reservoir (RHR), Jin-Sha Reservoir (JSR-K), Tian-Pu Reservoir (TPR), Lan-Hu Reservoir (LHR), Lian-Hu Reservoir (LianHR), Ling-Hu Reservoir (LingHR), Yang-Ming-Hu Reservoir (YMHR), Xi-Hu Reservoir (XHR), Jin-Hu Reservoir (JHR), and Tai-Hu Reservoir (THR) in Kinmen.

Table S2 – Monitoring results of *Microcystis* and microcystins for the samples collected from Tai-Hu Reservoir (THR).

Samples	Date		Uniplex (Ct value)		Duplex (Ct value)		MCs concentration (μg/L)
			*mcy*B	16S rRNA	*mcy*B	16S rRNA	
THR	2013	Feb.	36.99 (±0.12)[a]	37.52 (±0.17)	36.41 (±0.17)	37.10 (±0.27)	--
		May	34.93 (±0.07)	34.02 (±0.02)	35.06 (±0.17)	34.64 (±0.06)	0.37
		Aug.	--[b]	36.07 (±0.05)	--	34.67 (±0.06)	--
		Nov.	32.78 (±0.03)	29.99 (±0.05)	32.93 (±0.20)	28.90 (±0.09)	0.63
	2014	Mar.	--	--	--	--	--
		May	35.37 (±0.25)	34.49 (±0.25)	35.50 (±0.15)	33.50 (±0.19)	0.15
		July	33.57 (±0.02)	30.29 (±0.17)	33.50 (±0.18)	29.23 (±0.06)	0.52
		Dec.	36.48 (±0.44)	35.12 (±0.01)	36.76 (±0.17)	33.78 (±0.02)	--
	2015	Mar.	--	37.33 (±0.10)	--	36.11 (±0.16)	--
		Jun.	35.68 (±0.19)	35.93 (±0.15)	35.82 (±0.29)	34.54 (±0.08)	1.15
		Aug.	--	36.43 (±0.12)	--	35.18 (±0.05)	0.21
	2016	May	29.97 (±0.19)	27.20 (±0.16)	29.99 (±0.23)	26.30 (±0.33)	--
		Aug.	34.92 (±0.05)	35.03 (±0.12)	34.98 (±0.06)	33.75 (±0.06)	0.37

[a] ( ) represents standard deviation of 2 replicates.

[b] — represents the result < detection limit (Table S1 (SI)).

Table S3 – Monitoring results of *Cylindrospermopsis* and cylindrospermopsin for the samples collected from Tai-Hu Reservoir (THR).

Samples	Date		Uniplex (Ct value)		Duplex (Ct value)		CYN concentration (μg/L)
			pks	*rpo*C1	*pks*	*rpo*C1	
THR	2013	Feb.	38.64 (±0.14)	27.41 (±0.10)	--	28.48 (±0.06)	0.65
		May	36.18 (±0.21)	31.51 (±0.18)	35.64 (±0.38)	32.64 (±0.05)	1.49
		Aug.	35.78 (±0.11)	32.58 (±0.52)	35.16 (±0.17)	33.74 (±0.20)	1.72
		Nov.	37.13 (±0.17)	28.27 (±0.17)	--	29.26 (±0.05)	1.89
	2014	Mar.	35.68 (±0.15)	25.91 (±0.05)	--	27.10 (±0.04)	2.18
		May	33.39 (±0.11)	28.87 (±0.42)	32.73 (±0.07)	30.08 (±0.18)	3.01
		July	--	--	--	--	--
		Dec.	--	31.10 (±0.24)	--	32.20 (±0.07)	0.16
	2015	Mar.	--	35.05 (±0.04)	--	35.84 (±0.16)	0.51
		Jun.	--	30.38 (±0.09)	--	31.36 (±0.04)	0.52
		Aug.	--	32.07 (±0.06)	--	33.07 (±0.13)	--
	2016	May	--	--	--	--	--
		Aug.	34.34 (±0.15)	32.07 (±0.19)	33.68 (±0.11)	33.27 (±0.14)	0.79

[a] ( ) represents standard deviation of 2 replicates.

[b] — represents the result < detection limit (Table S1 (SI)).

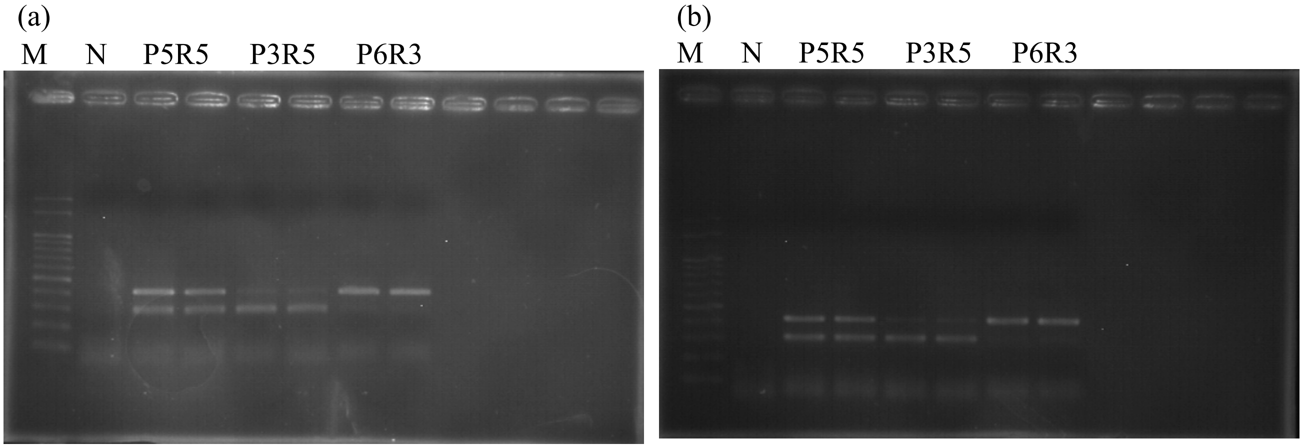

Figure S2 – Tests of inhibition on gene detection caused by different amounts of standard DNA using gel electrophoresis, where M represents the DNA marker, N represents the negative control, P5R5, P3R5 and P6R3 represent the concentration of *pks* gene and *rpo*C1 gene with 2 replicates, respectively (P5R5 = 10^5 and 10^5; P3R5 = 10^3 and 10^5; P6R3 = 10^6 and 10^3). (a) is for the duplex qPCR system with primer and probe sets of *pks* gene and *rpo*C1 gene; (b) is for the duplex qPCR system with primer sets of *pks* gene and *rpo*C1 gene (without probes).

Table S4 – The influence of primer concentration on the inhibition of gene detection.

Primer concentration		Duplex	
		pks gene	*rpo*C1 gene
0.1 μM	P6+R3 [3]	21.71 (±0.02)[1]	- [2]
0.1 μM	P3+R5	-	26.45 (±0.11)
0.2 μM	P6+R3	21.55 (±0.11)	-
0.2 μM	P3+R5	-	26.51 (±0.13)
0.3 μM	P6+R3	21.79 (±0.42)	-
0.3 μM	P3+R5	-	26.65 (±0.18)
0.4 μM	P6+R3	22.07 (±0.01)	-
0.4 μM	P3+R5	-	26.92 (±0.10)

[1] ( ) represents standard deviation of 2 replicates; [2] — represents the result < detection limit (Table S1 (SM)).

[3] P6R3 represent the concentration of *pks* gene and *rpo*C1 gene, respectively (P6R3 = 10^6 and 10^3).

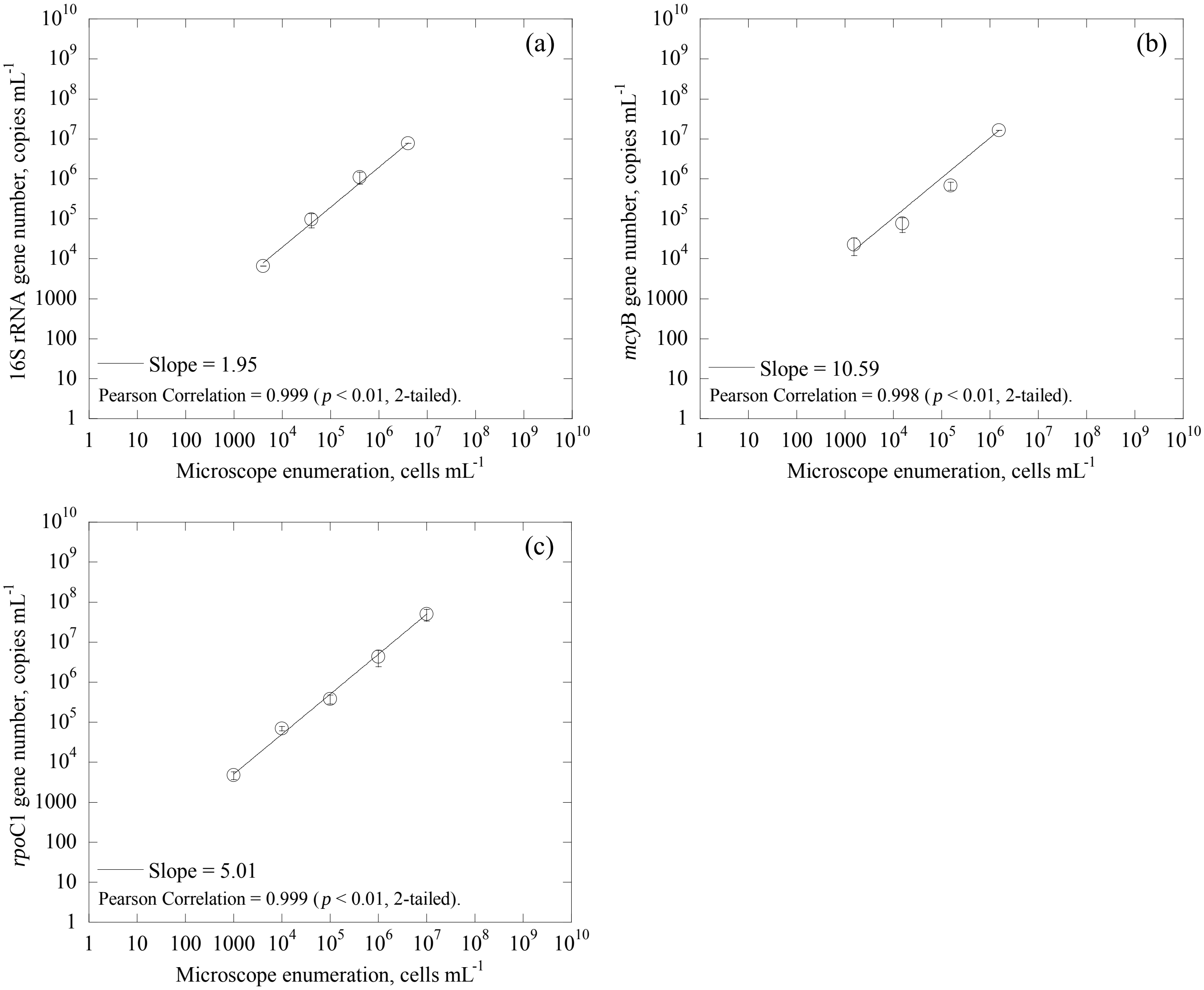

Figure S3 – The relationship between cell enumeration measured with microscopy and gene copy number with qPCR, where (a) is for 16S rRNA gene, (b) is for *mcy*B gene, and (c)is for *rpo*C1 gene. Error bars represent standard deviation of 2 replicates.

Table S5 – Correlation between MCs/CYN concentrations and cell equivalents.

Sample location	Data size	Correlation [e]		R^2	Pearson Correlation
Taiwan main island [a]	44	cell abundance vs 16S rDNA:	y = 0.750 + 0.741 x	0.749	0.786**
	22	MCs vs *mcy*B:	y = -0.661 + 0.381 x	0.690	0.831**
	7	cell abundance vs *rpo*C1:	y = 0.667 + 0.861 x	0.777	0.881**
	6	CYN vs *pks*:	y = -0.285 + 0.183 x	0.618	0.786*
Kinmen islands [b]	86	cell abundance vs 16S rDNA:	y = 1.070 + 0.710 x	0.769	0.877**
	38	MCs vs *mcy*B:	y = -0.650 + 0.354 x	0.731	0.855**
	91	cell abundance vs *rpo*C1:	y = 1.753 + 0.638 x	0.528	0.727**
	43	CYN vs *pks*:	y = -0.059 + 0.109 x	0.224	0.474**
Matsu islands [c]	43	cell abundance vs 16S rDNA:	y = 1.515 + 0.605 x	0.566	0.753**
	27	MCs vs *mcy*B:	y = -0.842 + 0.444 x	0.620	0.788**
	11	cell abundance vs *rpo*C1:	y = 0.513 + 0.814 x	0.792	0.890**
	4	CYN vs *pks*:	y = -0.232 + 0.172 x	0.880	0.938*
All data [d]	173	cell abundance vs 16S rDNA:	y = 1.002 + 0.713 x	0.740	0.860**
	87	MCs vs *mcy*B:	y = -0.671 + 0.374 x	0.683	0.827**
	109	cell abundance vs *rpo*C1:	y = 1.169 + 0.753 x	0.659	0.812**
	53	CYN vs *pks*:	y = -0.157 + 0.142 x	0.392	0.626**

a the data sizes were collected from 10 drinking water reservoirs (DWRs) in Taiwan main island.

b the data sizes were collected from 10 DWRs in Kinmen islands.

c the data sizes were collected from 9 DWRs in Matsu islands.

d the data sizes were collected from 29 studied DWRs in three areas.

e y is log(cell abundance/toxin concentration+1) and x is log(cell equivalents/gene copy+1).

* the pearson correlation is significant at the 0.1 level (2-tailed).

** the pearson correlation is significant at the 0.01 level (2-tailed).
